# Exploring the supportive care needs of families affected by pancreatic cancer: a mixed-methods study protocol

**DOI:** 10.1186/s12885-024-13335-x

**Published:** 2024-12-18

**Authors:** Tara Anderson, Gillian Prue, Lisa Graham-Wisener, Susan McLaughlin, Gary Mitchell

**Affiliations:** 1 School of Nursing and Midwifery, Queen’s University Belfast, Belfast, UK; 2 School of Psychology, Queen’s University Belfast, Belfast, UK; 3 Northern Ireland Pancreatic Cancer, Belfast, UK

**Keywords:** Pancreatic Cancer, Caregivers, Family, Psychosocial, Supportive Care Needs, Wellbeing, Quality of Life

## Abstract

**Background:**

Pancreatic cancer is an aggressive disease with most cases diagnosed at an advanced stage resulting in low survival rates. Family members often take on a role of supporting patients’ needs. Families tend to be unprepared for this and experience high levels of unmet needs and substantial impacts to their own wellbeing, heightened by the rapid deterioration and short life expectancy associated with pancreatic cancer.

**Aim:**

The proposed study aims to explore the supportive care needs and associated psychosocial impact of pancreatic cancer on family members, and the role of support services in supporting these families.

**Methods:**

A sequential explanatory mixed methods design will be utilised. Data collection will consist of three phases: (1) Survey of affected family members to explore their supportive care needs and psychological wellbeing; (2) Semi-structured interviews to explore the lived experiences of family members across the disease trajectory, their psychosocial adjustment, and their perceptions of support services; (3) Focus groups with support services providers to explore their experiences in providing support to affected families.

**Discussion:**

By combining quantitative and qualitative approaches, this research aims to provide a comprehensive understanding of the challenges and opportunities in providing psychosocial support to families affected by pancreatic cancer, ultimately enhancing their quality of life during and after the cancer journey. The findings may help to inform the development and enhancement of support programs, tailored to meet the specific needs of affected families.

## Background

Pancreatic cancer is an aggressive disease with a high mortality rate, often diagnosed at an advanced stage [1–3]. As the 11th most common cancer in the world, and the seventh leading cause of cancer deaths [4], pancreatic cancer represents a significant public health concern worldwide. Five-year survival rates are estimated to range from 5 to 15% [5], and unlike most common cancers which have seen decreased mortality rates over the past 25 years, the incidence and mortality rates of pancreatic cancer are continuing to increase [6, 7]. People living with pancreatic cancer often experience severe symptoms, report high levels of physical and psychological distress and experience a reduced quality of life (QoL) [8–10].

Family members often take on a role of supporting patients’ needs and become involved in tasks such as medication and symptom management [11]. Families tend to be unprepared for such a role and experience significant impact to their own psychological wellbeing and quality of life [12–14]. A cancer diagnosis can have a significant psychosocial impact on the patient’s family [15, 16]. This may include shock and denial at the time of diagnosis, and anxiety and/or depression at later stages [17]. For those supporting someone with pancreatic cancer, these impacts may be heightened by the rapid deterioration associated with the disease [18]. In particular, pancreatic cancer caregivers have been shown to experience a high prevalence of unmet needs [19, 20] and are more at risk of several psychiatric disorders compared to caregivers of most other cancers [21]. Pancreatic cancer presents unique challenges such as diet restrictions and pancreatic exocrine insufficiency which may add to the difficulties experienced by family caregivers [11, 13, 22].

The proposed study aims to add to existing literature by exploring the relationship between unmet needs and psychological outcomes to identify areas of need which, if addressed, may help lead to less negative emotional experiences. A scoping review, conducted by the authors, has identified a lack of research focused specifically on pancreatic cancer caregivers with the majority of existing studies including patients and/or caregivers of other cancer patients [23]. Due to the unique challenges presented by pancreatic cancer discussed, this is an important population to place particular focus on. In addition, pancreatic cancer caregivers consistently reported a lack of support throughout the literature included in this scoping review [23], highlighting the need to explore perceptions of support from both families affected by pancreatic cancer, and of those providing this support.

Fletcher et al. (2012) have synthesised previous cancer caregiving experience research into a conceptual model, the ‘Cancer Family Caregiving Experience’, consisting of three main elements: the stress process, contextual factors and the cancer trajectory [24]. Within this model, a stress process is proposed based upon the Transactional Model of Stress and Coping [25] consisting of five domains: primary stressors, secondary stressors, appraisal, cognitive and behavioural responses, health and wellbeing. Contextual factors (e.g. economic and cultural), and the cancer trajectory (e.g. diagnosis, treatment, survivorship, and bereavement) are also considered. This model will guide the proposed study, serving as an underpinning theoretical lens to explore the family experience of pancreatic cancer. Within this study, particular focus will be placed on the domains ‘appraisal’ and ‘cognitive-behavioural responses’ as these relate to how individuals appraise their needs and the extent to which these are met, and the actions they take in response to this. Appraisal and cognitive and behavioural responses are suggested to be the aspects of the caregiving experience which may be most amenable to intervention [24] and so these may be especially important to consider in order to inform the development and enhancement of support services for affected families. In line with this, the proposed study will place particular focus on ‘appraisal’ in terms of unmet needs, and ‘cognitive-behavioural responses’ in terms of the psychosocial adjustment of family members.

The proposed study will consist of three phases of data collection. First, a survey of family member supportive care needs and psychological outcomes. This will explore the relationship between their unmet needs, an aspect of appraisal, and health and wellbeing outcomes. Secondly, follow-up interviews with participants will enable the exploration of these factors in relation to other aspects of the model. In particular, cognitive behavioural responses will be explored in relation to the psychosocial adjustment of family members. Finally, focus groups with support service providers will enable exploration of the experiences of support service providers, and the barriers and facilitators they face in supporting these families. Overall, this study aims to explore the supportive care needs of families affected by pancreatic cancer and the role of support services via the following objectives:

1. Explore the supportive care needs and psychological wellbeing of families affected by pancreatic cancer.
2. Explore the psychosocial adjustment of family members across the disease trajectory.
3. Gain an insight into the availability and utilisation of support services from the perspective of families affected by pancreatic cancer.
4. Explore the experiences of support services providers and consider the facilitators and barriers in providing timely psychosocial support to families affected by pancreatic cancer.

## Methods

### Expert reference group

The board members of Northern Ireland Pancreatic Cancer (NIPANC), a charity working to improve the outcomes of pancreatic cancer, will occupy the role of expert reference group for the proposed research. The expert reference group have been involved in the development of this proposal and will continue to guide the research process going forward.

The nine board members are individuals with professional, and/or personal experience of pancreatic cancer. This includes two pancreatic cancer survivors, six individuals who lost a loved one to pancreatic cancer, and a consultant hepatobiliary surgeon.

### Design

**Fig. 1:**
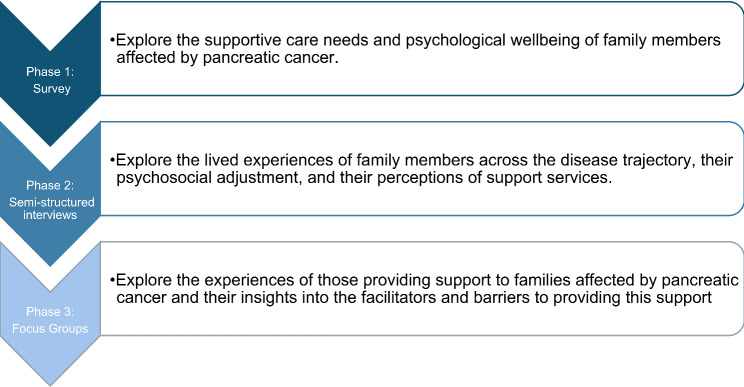
Study Overview

This study will employ a mixed-methods approach following a sequential explanatory design (Fig. 1) consisting of three phases: (1) Survey of affected family members; (2) Semi-structured interviews with family members; (3) Focus groups with support services providers.

### Phase 1

A survey will be administered to family members of those living with a pancreatic cancer diagnosis. This survey aims to explore the supportive care needs and psychological wellbeing of family members affected by pancreatic cancer, the relationship between these, and the relationship between participant characteristics and areas of unmet need.

### Participants and recruitment

Participants will be a family member of someone currently living with a pancreatic cancer diagnosis at any stage of the disease trajectory (including those regarded as survivors). Family members who have been bereaved will not be eligible to participate in this survey as they will be asked about current unmet needs in relation to the care they are currently providing for the person with cancer. Full eligibility criteria are presented in Table 1. The survey will be advertised via links to the online survey on social media and via the expert reference group’s networks. The survey will begin with two screening questions (are you over 18 years of age and is your loved one currently living with a pancreatic cancer diagnosis) to ensure participants meet the eligibility criteria. If potential participants select ‘no’ to either screening question, they will be re-directed to appropriate support services.

**Table 1:** Eligibility criteria for phase one

Inclusion criteria	Exclusion criteria
A family member of someone with a previous pancreatic cancer diagnosis (this may include partners, siblings, adult children, parents)	Family members who have been bereaved as a result of pancreatic cancer
A family member to someone with a pancreatic cancer diagnosis at any disease stage including those regarded as survivors or undergoing surveillance.	Individuals who are under 18 years of age

### Data collection

The survey will be administered online via Microsoft Forms. Paper copies of the survey will also be available on request. The survey consists of 85-items in total. First, ten demographic questions will be presented. These will include age group, gender, ethnicity, NI health and social care trust area (for those living in NI), highest level of education, and a measure of subjective socioeconomic status. Details regarding the participant’s relationship to the person with pancreatic cancer and the person with cancer’s disease status will also be collected.

The survey will then consist of four questionnaires: (1) The Supportive Care Needs Survey for Partners and Carers (SCNS-P&C), (2) The Generalised Anxiety Disorder questionnaire (GAD-7), (3) The Patient Health questionnaire (PHQ-9), and (4) The Warwick-Edinburgh Mental Wellbeing Scale (WEMWBS). The SCNS-P&C will provide an assessment of the levels of unmet needs for caregivers. The following three measures will provide a holistic view of caregiver’s psychological wellbeing as both the GAD-7 and PHQ-9 focus on negative symptoms while the WEMWBS covers more positive aspects of mental health.

### Measures

The MacArthur Scale of Subjective Social Status [26] will be utilised to measure subjective socioeconomic status. Participants are presented with a picture of a ladder with 10 rungs with the top of the ladder representing the people who are the best off (have the most money, most education and most respected jobs) and the bottom of the ladder representing those who are the worst off (have the least money, least education, least respected jobs). Participants will be asked to choose the section they would place themselves on this ladder relative to other people in society. This a validated item with robust predictive validity [27] and has shown to be valid measure when correlated with objective measures of socioeconomic status [28].

The Supportive Care Needs Survey for Partners and Carers (SCNS-P&C) [29] is a 45-item questionnaire designed to provide a comprehensive assessment of the multi-dimensional supportive care needs of cancer caregivers. The measure contains four domains: healthcare service needs (10 items), psychological and emotional needs (14 items), work and social needs (7 items), information needs (8 items), and other (6 items). Participants rate each item from one (no unmet need) to five (high unmet need). Higher scores on each subscale represent increased unmet needs in this area. Previous research has established good internal validity of these domains with Cronbach’s alpha ranging from 0.88 to 0.94 [29]. This measure has also shown satisfactory face, content, and construct validity across various languages and cultures with caregivers of cancer patients [30–33]. Additionally, this scale has been used previously with caregivers of people with pancreatic cancer [19, 20].

The Generalised Anxiety Disorder (GAD-7) questionnaire is a seven-item measure designed to screen for GAD and assess symptom severity based on the Diagnostic and Statistical Manual of Mental-Disorders-IV (DSM-IV) [34]. Each item (e.g. “Over the last 2 weeks, how often have you been bothered by worrying too much about different things?”) is scored on a four-point Likert scale from zero (none) to three (almost every day). Scores range from zero to 21, with scores of zero to four interpreted as mild, five to nine as moderate, 10–14 as high, and 15–21 as severe. Previous research has established high internal consistency and test-retest reliability of this scale [34].

The Patient Health Questionnaire (PHQ-9) is a nine-item scale based on the DSM-IV for major depressive disorder [35]. Each item (e.g. “Over the last 2 weeks, how often have you been bothered by feeling down, depressed, or hopeless?”) is scored on a four-point Likert scale from zero (none) to three (almost every day). Scores range from zero to 27, with scores of zero to four interpreted as minimal, five to nine as mild, 10–14 as moderate, 15–19 as high, and 20–27 as severe. This measure has shown excellent internal consistency and test-retest reliability [35]. Both the GAD-7 and PHQ-9 have been used together in previous studies with cancer caregivers [36–38].

The Warwick-Edinburgh Mental Well-Being Scale (WEMWBS) [39] is a measure of mental wellbeing focused on positive aspects of mental health (e.g. “I’ve been feeling optimistic about the future”). It is a 14-item scale scored from 14 to 70 with higher scores representing higher levels of wellbeing. Each item is scored on a scale from one (none of the time) to five (all of the time). This scale shows high levels of internal consistency and reliability [39] and has been used globally with different populations including cancer caregivers [40].

### Data analysis

Two multivariate multiple regression analyses will be conducted to examine: (1) the relationship between demographic details and areas of unmet supportive care needs, and (2) the association between unmet supportive care needs and levels of anxiety, depression and wellbeing. The ten demographic details collected will act as predictor variables within the first regression model to explore the association between each characteristic and areas of unmet need. Next, each of the five domains of the SCNS-P&C will act as a predictor variable within a second regression model to explore the association between each domain and psychological outcomes.

As the first regression model includes the greatest number of predictor variables, this will require the larger sample size. An a priori power analysis was conducted using G*Power version 3.1.9.6 [41] to determine the minimum sample size required for this regression analysis. Results indicated the required sample size to achieve 80% power for detecting a medium effect, at a significance criterion of *α* = 0.05, was *N* = 214. Therefore, at least 214 participants will be recruited for phase one.

### Phase 2

A sub-sample of family members who have completed the survey will be recruited to participate in a semi-structured interview exploring their experiences of supporting someone with pancreatic cancer. The interviews will explore family member’s lived experiences across the disease trajectory, psychosocial adjustment, and their perceptions of support services.

### Participants and recruitment

Survey participants will be asked to provide contact details if they wish to be contacted about completing a follow-up interview regarding their experiences. Therefore, the eligibility criteria for phase two are the same as that presented for phase one (Table 1). However, due to the nature of pancreatic cancer, it is likely some participants from phase one may have lost their loved one between their survey response and potential interview participation. Therefore, bereaved family members may be included in phase two interviews, if these participants wish to continue their participation in the study. Approximately 15–20 participants will be recruited for phase two. This is an estimate, guided by information power [42]. Participants for this phase will be recruited using convenience sampling based on a first-come, first-served basis of those who volunteer. However, depending on response rates, purposive sampling may be utilised to cover a range of stages of the disease trajectory.

According to the information power model for qualitative interview studies, a smaller sample size is recommended for studies in which participant characteristics are specific to the study aim, the study is supported by established theory, and the analysis includes in-depth exploration of narrative details [42]. Therefore, the proposed study may require a smaller sample size due to the sampling of participants with specific characteristics, application of the ‘Cancer Family Caregiving Experience’ conceptual model [24], and exploratory in-depth analysis of narratives. Although the broad study aim, to explore experiences across the disease trajectory, may require an increased sample size. Therefore, the recruitment target is an estimate based on these recommendations and the experience of the research team which will be reviewed throughout the process.

### Data collection

Interviews will be guided by the ‘Cancer Family Caregiving Experience’ conceptual model [24] to explore the lived experience of the family member across their loved one’s disease trajectory and obtain greater insights into the quantitative results gained in phase one. In addition, interviews will explore the psychosocial adjustment of family members to include their psychosocial and emotional experiences, and cognitive and behavioural responses to these. Finally, interviews will explore the perceived availability, utilisation, and impact of support services. This may include experiences of support provided via the patient’s care team and engagement with external organisations such as counselling services.

An interview guide will be used flexibly and developed based on phase one results. A distress protocol will be followed to ensure participants are supported if they become distressed, this will include signposting to support services and the opportunity to stop or pause participation.

### Data analysis

With participant’s consent, interviews will be audio-recorded, transcribed verbatim, and analysed using reflexive thematic analysis [43]. Data will be managed and coded using NVivo qualitative data analysis software.

A deductive approach to reflexive thematic analysis will be utilised, as the ‘Cancer Family Caregiving Experience’ conceptual model [24] will provide a lens through which the qualitative data will be analysed and interpreted. This will involve considering data in line with the model but remaining open to new insights emerging from the data [43]. The research team will critically reflect on how the existing model influences their interpretations of the data.

### Phase 3

Focus groups will be conducted with support service providers to explore their experiences in providing support to families affected by pancreatic cancer and their insights into the facilitators and barriers to providing this support.

### Participants and recruitment

Approximately 3–4 focus groups, each comprising of 5–6 support service providers will be held. Participants will be those who provide support, in a professional or voluntary capacity, to families affected by pancreatic cancer. Full eligibility criteria is presented in Table 2. Support service providers may be recruited based on the support services identified by participants in phase two, and as recommended by the expert reference group (NIPANC). This is expected to include, for example, cancer nurse specialists, counselling and support staff from local charities such as Cancer Focus NI, Macmillan Cancer Support, and Marie Curie.

**Table 2:** Eligibility criteria for phase three

Inclusion criteria
Individuals who provide support, in a professional or voluntary capacity, to family members of those living with a pancreatic cancer diagnosis.
At least 18 years old.
Participants must have been in their current support role for at least six months.

### Data collection

Focus groups will be held in person or may be conducted online (via Microsoft Teams) to aid convenience for participants. Focus group participants will complete a short demographic survey via MS Forms regarding their characteristics (age group, gender, ethnicity) and experience (role, sector, length of experience) prior to participation. A focus group guide will be developed based on the results of the previous phases and used flexibly to guide discussion.

### Data analysis

With participants’ consent, focus groups will be audio-recorded, transcribed verbatim, and analysed using reflexive thematic analysis [43]. Focus group data will be managed and coded using NVivo qualitative data management software.

### Data integration

In line with a sequential explanatory mixed-methods design, each phase will inform the next [44, 45]. Therefore, survey data will be collected first and used to inform the development of the interview guide for phase two. Additionally, depending on response rates and the number of volunteers for phase two, interview participants may be sample purposively based on their loved one’s stage in the disease trajectory, as indicated in the survey. Focus groups will be conducted last and the guide used to facilitate these will be informed by the results of both the survey and the interviews.

Data from all phases will then be taken together to provide a comprehensive view of the unmet needs of families affected by pancreatic cancer, and the challenges and opportunities to supporting these families. Data from all phases will be integrated utilising the ‘following a thread’ framework [46]. This involves the initial analysis of one dataset to identify areas requiring further exploration and following this across phases to generate a multi-faceted view of the explored phenomenon [46, 47]. This approach has been suggested to help preserve the integrity of each individual dataset while providing an opportunity to consider all data together to generate an overall picture of findings [48].

Within the proposed study, this framework will be applied to data integration during development of the interview guide and focus group guide, and to collectively interpret data from all phases following completion of the final phase. Data from each phase will first be analysed separately and used to inform the next phase (via interview or focus group guide development). Finally, areas of interest identified from each phase (i.e. threads) will be explored throughout all phases. For example, if a particular area of unmet need is identified in the survey this may be explored further with interview participants to identify their perceptions of this, and further with support service providers in focus groups to explore how they perceive this need may be met. Likewise, a barrier to providing support identified in focus groups may be traced back to participant’s perceptions of support discussed in interviews.

### Reflexivity

Qualitative data from phases two and three will be analysed using reflexive thematic analysis. Reflexivity, the influence of the researcher’s background and prior perceptions on the research process, is an important consideration of Braun and Clarke’s six-phase framework for reflexive thematic analysis [43]. Therefore, to acknowledge the impact of any potential biases, the research team will collaboratively discuss their preconceptions and assumptions which may affect the analysis process, prior to beginning analysis. Additionally, transcriptions will be completed by one member of the research team and verified by a second member. A reflexive journal will be kept to document thoughts, decisions, and interpretations of the researcher completing the initial coding. Finally, all themes will be discussed and developed collaboratively by the research team.

### Ethical and governance issues

This study received ethical approval from the Faculty of Medicine, Health and Life Sciences Research Ethics Committee at Queen’s University Belfast in October 2024 (Reference: 24_137).

#### Consent to participate

will be gathered from all participants (affected family members, and support service providers). All participants will be informed that their participation in the study is entirely voluntary, and they can choose to withdraw from the study at any time prior to or during data collection.

As participation may involve participants discussing emotional issues in regard to their experiences, the researcher will follow a distress protocol during interviews and focus groups. This will involve allowing participants to move past a question they do not wish to answer, asking participants if they would like to end their participation temporarily or permanently if they become distressed, and signposting to relevant support services. In addition, a support services resource leaflet has been incorporated into each phase of the study to ensure all potential participants have access to this information.

## Discussion

Quantitative findings will provide an insight into the prevalence of unmet supportive care needs, and psychological outcomes within families affected by pancreatic cancer. This may include identifying characteristics which may be associated with particular areas of unmet need as well as the impact of different areas of unmet need on psychological outcomes. Qualitative findings, first from follow-up interviews with family members, will provide further insight into the lived experiences of those supporting a loved one with pancreatic cancer. This phase will enable exploration of the factors identified in the survey to better understand how specific areas of unmet need impact family members and their psychosocial adjustment across the disease trajectory. Interviews will also identify the support that family members engage with and their perceptions of available support. This will provide an insight into existing support for families affected by pancreatic cancer which will be further explored within the focus groups with support service providers. Focus group findings will provide a greater understanding of the experiences of those supporting families and their perceptions of how this support may be enhanced.

The National Institute for Health and Care Excellence (NICE) recommends providing individuals diagnosed with pancreatic cancer and their family members with information and support to help them manage the psychological impact of the disease on their lives [49]. The proposed research may help to inform this, leading to enhanced support relevant to the stage of disease and tailored to specific needs as recommended by NICE guidelines [49]. Additionally, the launch of the optimal care pathway for pancreatic cancer in the UK which aims to implement better standards of care for people with pancreatic cancer, provides a timely opportunity for the enhancement of support services for families [50].

Taken together, the findings of this study will inform the development and enhancement of support programs, tailored to meet the specific needs of affected families. Moreover, this research will foster collaboration between healthcare providers, statutory services, and charities, ultimately improving the well-being of families dealing with pancreatic cancer. By combining quantitative and qualitative approaches, this research aims to provide a comprehensive understanding of the challenges and opportunities in providing psychosocial support to these families, ultimately enhancing their quality of life during and after the pancreatic cancer journey.

## Data Availability

No datasets were generated or analysed during the current study.
